# JDP2: An oncogenic bZIP transcription factor in T cell acute lymphoblastic leukemia

**DOI:** 10.1084/jem.20170484

**Published:** 2018-07-02

**Authors:** Marc R. Mansour, Shuning He, Zhaodong Li, Riadh Lobbardi, Brian J. Abraham, Clemens Hug, Sunniyat Rahman, Theresa E. Leon, You-Yi Kuang, Mark W. Zimmerman, Traci Blonquist, Evisa Gjini, Alejandro Gutierrez, Qin Tang, Laura Garcia-Perez, Karin Pike-Overzet, Lars Anders, Alla Berezovskaya, Yi Zhou, Leonard I. Zon, Donna Neuberg, Adele K. Fielding, Frank J.T. Staal, David M. Langenau, Takaomi Sanda, Richard A. Young, A. Thomas Look

**Affiliations:** 1 Department of Haematology, University College London Cancer Institute, London, England, UK; 2 Department of Pediatric Oncology, Dana-Farber Cancer Institute, Harvard Medical School, Boston, MA; 3 Molecular Pathology and Cancer Center, Massachusetts General Hospital, Boston, MA; 4 Harvard Stem Cell Institute, Stem Cell and Regenerative Biology Department, Harvard University, Cambridge, MA; 5 Whitehead Institute for Biomedical Research, Cambridge, MA; 6 Heilongjiang River Fisheries Research Institute of Chinese Academy of Fishery Sciences, Harbin, China; 7 Department of Biostatistics and Computational Biology, Dana-Farber Cancer Institute, Boston, MA; 8 Stem Cell Program and Division of Hematology/Oncology, Boston Children’s Hospital and Dana Farber Cancer Institute, Howard Hughes Medical Institute, Harvard Medical School, Boston, MA; 9 Department of Immunohematology, Leiden University Medical Center, Leiden, Netherlands; 10 Cancer Science Institute of Singapore, National University of Singapore, Singapore; 11 Department of Medicine, Yong Loo Lin School of Medicine, Singapore; 12 Department of Biology, Massachusetts Institute of Technology, Cambridge, MA

## Abstract

A substantial subset of patients with T cell acute lymphoblastic leukemia (T-ALL) develops resistance to steroids and succumbs to their disease. *JDP2* encodes a bZIP protein that has been implicated as a T-ALL oncogene from insertional mutagenesis studies in mice, but its role in human T-ALL pathogenesis has remained obscure. Here we show that *JDP2* is aberrantly expressed in a subset of T-ALL patients and is associated with poor survival. JDP2 is required for T-ALL cell survival, as its depletion by short hairpin RNA knockdown leads to apoptosis. Mechanistically, JDP2 regulates prosurvival signaling through direct transcriptional regulation of *MCL1.* Furthermore, *JDP2* is one of few oncogenes capable of initiating T-ALL in transgenic zebrafish. Notably, thymocytes from *rag2:jdp2* transgenic zebrafish express high levels of *mcl1* and demonstrate resistance to steroids in vivo. These studies establish *JDP2* as a novel oncogene in high-risk T-ALL and implicate overexpression of *MCL1* as a mechanism of steroid resistance in *JDP2*-overexpressing cells.

## Abstract (precis)

Mansour et al. demonstrate that JDP2, a bZIP transcription factor, is overexpressed in patients with high-risk T cell acute lymphoblastic leukemia (T-ALL). JDP2 initiates T-ALL in a zebrafish model and leads to steroid resistance in vivo through direct regulation of MCL1.

## Graphical Abstract

**Figure:**
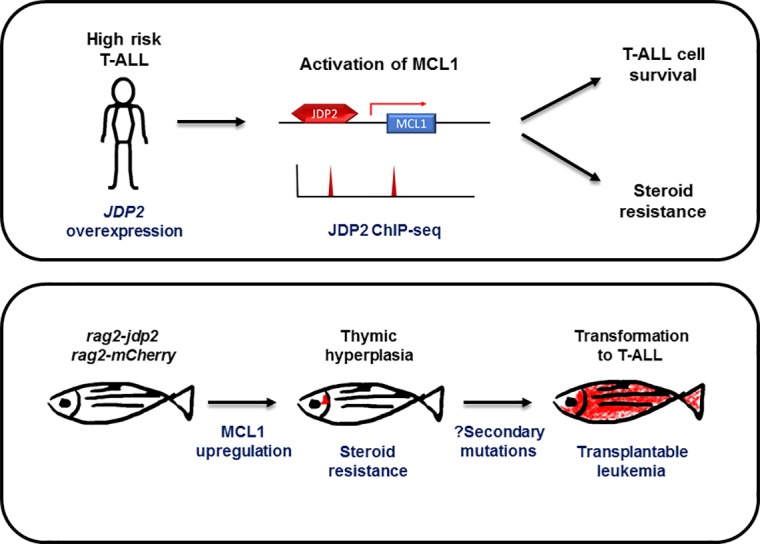

## Introduction

With the availability of next-generation sequencing technology, a large number of recurrently mutated genes have been identified that contribute to the molecular pathogenesis of cancer, spurring the development of targeted therapies. However, such large-scale genomic approaches also identify a significant number of “passenger” mutations of uncertain functional relevance and disregard potential oncogenes whose expression is dysregulated in the absence of an identifiable genomic lesion. A powerful complementary approach to the discovery of novel oncogenes and tumor suppressors has been the use of retroviral and transposase-based insertional mutagenesis screens (Nusse and Varmus, 1982; Callahan, 1996; Collier et al., 2005; Dupuy et al., 2005, 2006; Copeland and Jenkins, 2010; McIntyre et al., 2012). For instance, *int-2*/*fibroblast growth factor-3* (*FGF3*), an oncogene that is recurrently amplified in human breast tumors, first came to prominence because of its dysregulation resulting from a shared integration site of mouse mammary tumor virus in murine models of breast cancer (Nusse and Varmus, 1982; Casey et al., 1986; Callahan, 1996). Insertional mutagenesis screens have been particularly effective in identifying novel oncogenes in T cell acute lymphoblastic leukemia (T-ALL). The first studies implicating Notch-1 as a major driver of T-ALL came from insertional mutagenesis screens using Moloney murine leukemia virus injected into neonatal mice (Girard et al., 1996). Remarkably, the majority of insertions occurred within the HD and PEST domains of *Notch-1*, sites that are hotspots for somatic mutation in a high proportion of human T-ALL cases, highlighting the relevance of such approaches to human biology (Girard et al., 1996; Hoemann et al., 2000; Weng et al., 2004).

*JDP2* is a transcription factor whose expression is recurrently up-regulated because of a common integration site in murine insertional mutagenesis models of T-ALL, yet its role in the human disease has not been investigated (Stewart et al., 2007; Rasmussen et al., 2009, 2010). This small bZIP protein contains an N-terminal domain that recruits cofactors, a basic domain that binds DNA, and a leucine zipper domain capable of heterodimerization with other bZIP proteins, such as c-JUN and DDIT3 (Aronheim et al., 1997; Weidenfeld-Baranboim et al., 2008). The role of JDP2 in cancer is controversial because it can partially transform chicken embryonic fibroblasts and accelerate hepatocellular carcinoma in mice, yet it has a tumor-suppressor role in human prostate cancer, features that may relate to its ability to both activate and repress AP-1 target sites, depending on the cellular context and bZIP binding partner (Blazek et al., 2003; Heinrich et al., 2004; Bitton-Worms et al., 2010).

Here we show that *JDP2* is frequently aberrantly expressed in human T-ALL and establish its oncogenic role by demonstrating that it can initiate T-ALL in transgenic zebrafish. *JDP2* overexpression is associated with a poor outcome in patients and is required for survival of human T-ALL cells in vitro. Mechanistically, JDP2 transcriptional activity promotes cell survival through direct activation of the anti-apoptotic MCL1 protein. Finally, we show that *jdp2* overexpression leads to *mcl1* up-regulation and steroid resistance in vivo, providing a potential explanation for the poor survival of T-ALL patients whose leukemic blasts overexpress JDP2.

## Results

### Jdp2 is a common integration site in murine models of T-ALL

**Figure 1.**
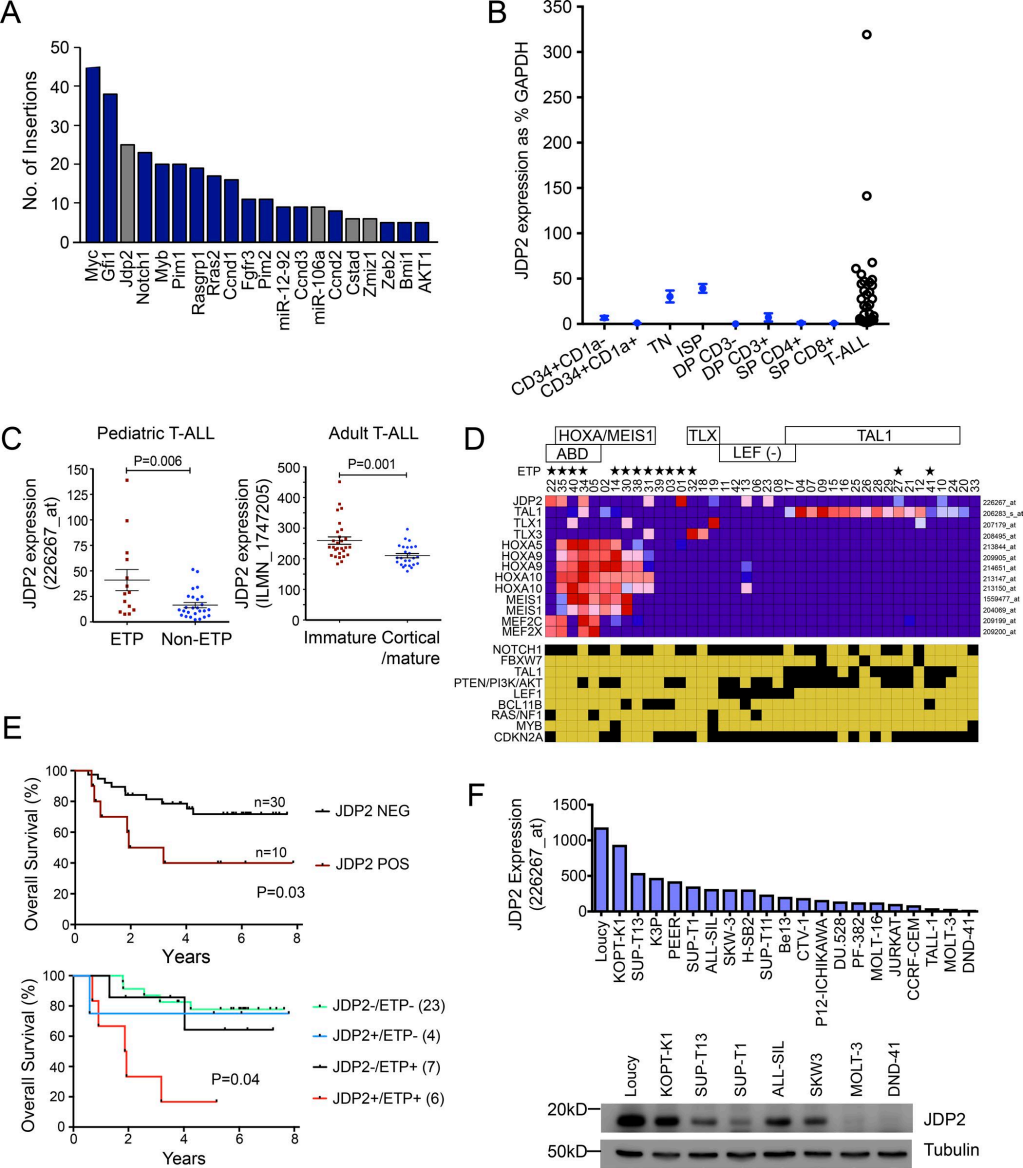
***JDP2* is a common integration site in murine insertional mutagenesis studies of T-ALL and is aberrantly expressed in some patients with T-ALL. (A)** Number of insertions identified from multiple murine retroviral insertional screens for T-ALL, collated on the RTCG database (Akagi et al., 2004). Gray bars are genes not yet implicated in human T-ALL. **(B)** *JDP2* mRNA expression as determined by qPCR from 34 diagnostic adult T-ALL cases from the UKALL14 trial (black circles) and directly compared with normal thymic subsets sorted by FACS (blue circles). Thymocyte subsets were pooled from five individual donors to reduce intersample variation. qPCR experiments were performed in triplicate from two independent experiments. TN, triple-negative; DP, double-positive; SP, single-positive. Data points represent the mean ± standard error of the mean. **(C)** *JDP2* expression as determined by Affymetrix gene expression array data for 40 pediatric T-ALL patients treated on the COG P9404 trial, separated according to ETP status (Gutierrez et al., 2010). *JDP2* expression as determined by Illumina bead-chip array for 53 adult T-ALL patients treated on the ECOG E2993 trial, comparing patients with immature versus cortical/mature phenotypes (Van Vlierberghe et al., 2013). P values were calculated using the two-tailed Student’s *t* test. **(D)** Heatmap showing Affymetrix gene expression data for T-ALL patients treated on the COG P9404 trial, together with the mutational status of recurrently mutated genes. Yellow boxes denote wild-type genes, and black boxes, the presence of a genetic lesion. **(E)** Kaplan–Meier curves showing overall survival for pediatric T-ALL patients treated on the COG P9404 trial, stratified by *JDP2* and ETP status. Patients were considered *JDP2*-positive using a probe presence score of ≥30; survival differences were calculated using the log-rank test. **(F)** *JDP2* gene expression in 21 human T-ALL cell lines as determined by Affymetrix gene expression array. Lower panel shows JDP2 protein expression in a selection of T-ALL cell lines as determined by Western blot analysis. Representative Western blot from two independent experiments is shown.

To identify novel human T-ALL oncogenes, we explored the Retrovirus and Transposon Tagged Cancer Gene Database (RTCGD), which contains the collated results of insertional mutagenesis studies of murine T-ALL (Akagi et al., 2004). The majority of recurrent retroviral integration sites were in the vicinity of genes with well-recognized roles in T-ALL pathogenesis, including (in order of frequency) *Myc*, *Gfi1*, *Notch1*, *Myb*, *Pim1*, *miR17-92*, *Ccnd3*, *Zeb2*, and *Akt1* (Fig. 1 A). Notably, *Jdp2*, encoding a bZIP protein without a reported role in human T-ALL, was the third most frequently retrovirally targeted gene, with the majority of insertions occurring on a CD2-*Myc*/*Runx2* genetic background, suggesting that *Jdp2* likely collaborates with these genes in transformation (Stewart et al., 2007). Insertions were clustered either within intron 2 or ∼50 kb upstream of the transcription start site (TSS), with most oriented antisense to *Jdp2* and reported to activate gene expression (Rasmussen et al., 2009, 2010). Insertions in the vicinity of *Jdp2* are not limited to retroviral models of T-ALL; recent studies of T-ALL initiated by the *Sleeping Beauty* transposon have also identified a shared integration site at the *Jdp2* promoter and have shown that the inserted transposon drives *Jdp2* overexpression (van der Weyden et al., 2013). Thus, both genome-wide retroviral and transposon insertional experiments implicate *Jdp2* as a T-ALL oncogene in mice.

### JDP2 expression is associated with early T cell progenitors (ETPs)/immature T-ALL and poor outcome

To address whether *JDP2* is aberrantly expressed in T-ALL patients compared with normal thymocytes, we isolated human thymic subsets at various levels of differentiation and performed quantitative RT-PCR (qPCR) for *JDP2*. A brief wave of *JDP2* expression was detected at the immature single-positive (ISP) and triple-negative stage of thymic maturation but was not detected in CD34^+^CD1a^−^ ETPs or more mature thymocytes. In direct comparison, 10 of 34 T-ALL patients expressed *JDP2* at higher levels than ISP cells (Fig. 1 B). Analysis of published datasets showed that the gene was more highly expressed in both pediatric and adult T-ALL patients whose leukemic cells had an immature/ETP-ALL phenotype (Fig. 1 C; P = 0.006 and P = 0.001, respectively), at a stage when *JDP2* is not normally expressed (Gutierrez et al., 2010; Van Vlierberghe et al., 2013). High levels of *JDP2* expression were not obviously associated with other specific genetic or cytogenetic lesions (Fig. 1 D; Gutierrez et al., 2010; Van Vlierberghe et al., 2013). Given previous studies that ETP-ALL is associated with a poor outcome (Coustan-Smith et al., 2009), we stratified patients according to *JDP2* expression level and analyzed their survival. *JDP2*-positive patients had an inferior probability of 5-yr overall survival compared with *JDP2*-negative patients (40% vs. 74% 5-yr overall survival, respectively, P = 0.03; Fig. 1 E), raising the possibility of a direct role for JDP2 in chemoresistance. In support of this concept, although patient numbers were small, *JDP2* was a poor prognostic marker within the ETP group itself, identifying an ETP^+^JDP2^+^ group with a particularly dismal outcome (Fig. 1 E).

To identify a cell line model to further investigate JDP2 function, we assessed *JDP2* mRNA expression in a panel of >20 human T-ALL cell lines. *JDP2* was most highly expressed in Loucy cells (Fig. 1 F), a cell line with the gene expression signature of ETP-ALL, which is consistent with data from primary patient samples (Van Vlierberghe et al., 2011; Anderson et al., 2014). Furthermore, JDP2 was highly expressed at the protein level in a subset of the T-ALL cell lines by Western blotting, at levels that correlated with mRNA expression levels (Fig. 1 F).

### JDP2 is required for the survival of T-ALL cells

**Figure 2.**
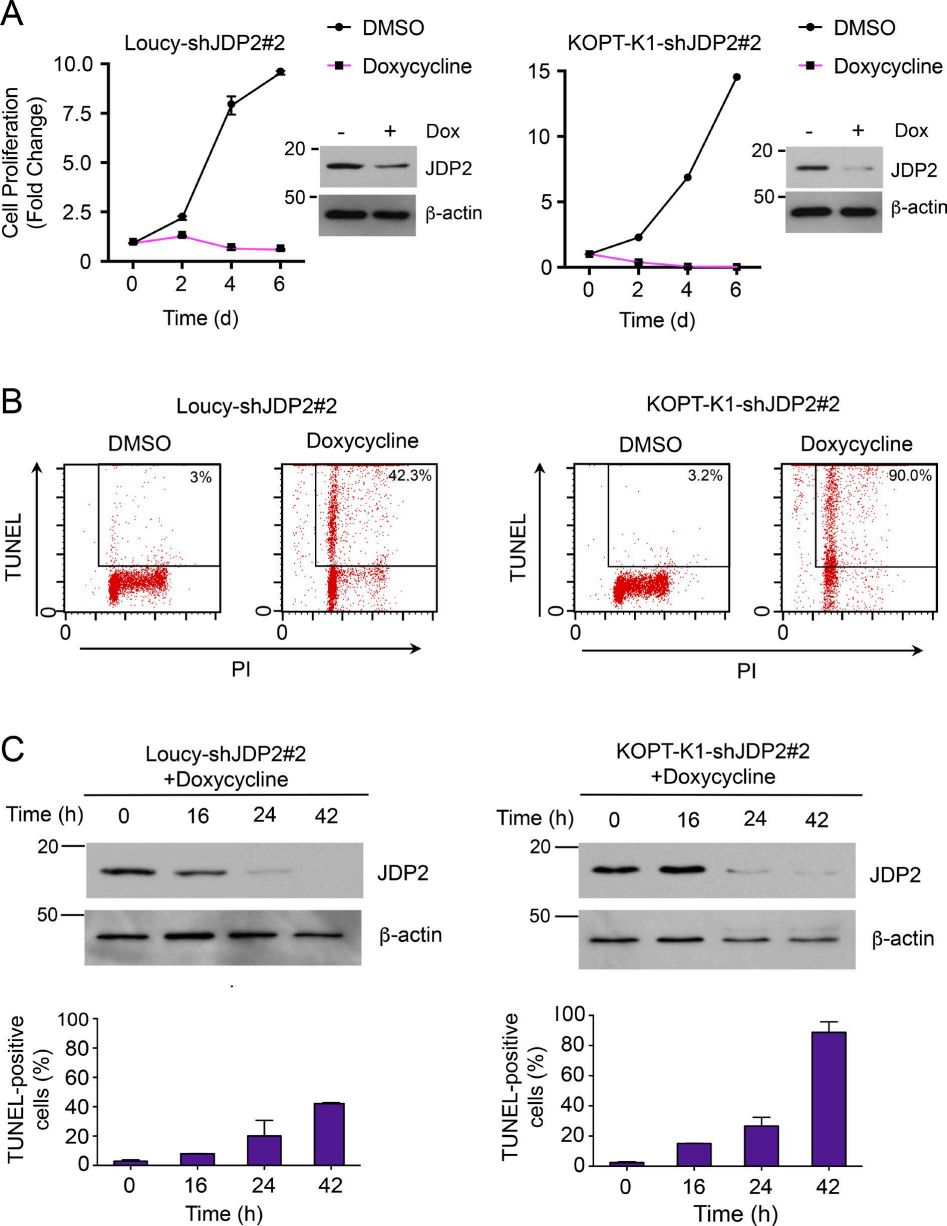
**Human T-ALL lines are highly dependent on JDP2 for cell growth. (A)** Cell growth kinetics in Loucy and KOPT-K1 T-ALL cells, stably expressing a doxycycline-inducible *JDP2* shRNA. Corresponding Western blots showing JDP2 protein expression with or without doxycycline. Representative data from three separate experiments are shown. **(B)** JDP2 depletion induces apoptosis in human T-ALL cell lines as determined by flow cytometric evaluation for TUNEL and PI in Loucy (left) and KOPT-K1 (right) cells stably expressing a doxycycline-inducible JDP2 shRNA, with and without doxycycline treatment for 24 h. Data were verified in two independent experiments. **(C)** Time course showing the kinetics of JDP2 depletion by Western blot analysis, and apoptosis induction measured by flow cytometry for TUNEL, in doxycycline-inducible JDP2 shRNA Loucy (left) and KOPT-K1 (right) cells after the addition of doxycycline. Representative Western blot from two independent experiments is shown. Data points represent the mean ± standard error of the mean.

To assess whether JDP2 is required for the survival of T-ALL cells, we performed shRNA knockdown experiments in Loucy and KOPT-K1 cells, the two T-ALL cell lines with the highest levels of JDP2 expression. We identified two independent shRNAs (shRNA#1 and #2) that efficiently depleted JDP2 protein expression, and both shRNAs significantly impaired growth of Loucy and KOPT-K1 cells (Fig. S1). The JDP2 shRNAs did not demonstrate broad toxicity across a panel of cancer cell lines as determined through the Achilles project (Fig. S2 A; Tsherniak et al., 2017), nor in DND-41 T-ALL cells that do not express JDP2 (Fig. S2 B). Importantly, we were able to completely rescue the inhibitory effects on cell growth through stable expression of a *JDP2* transgene containing “wobble” bases at the target site of shRNA#2, showing that the effect on cell growth was caused by knockdown of *JDP2* and not off-target shRNA activities (Fig. S2 C). To study the effects of JDP2 depletion in more detail, we established stable cell line clones expressing a doxycycline-inducible *JDP2* shRNA#2. shRNA induction led to a dramatic impairment of cell growth in both cell lines (Fig. 2 A). This effect was mediated through apoptosis as determined by flow cytometry for TdT-dUTP nick-end labeling (TUNEL) and annexin V (Fig. 2 B and Fig. S3 A), as well as PARP1 cleavage and cleaved caspase-3 (Fig. S3 B). Furthermore, the kinetics of the onset of apoptosis corresponded with JDP2 protein depletion (Fig. 2 C).

### JDP2 binds to TPA-response element (TRE) sites and can both activate and repress its target genes

**Figure 3.**
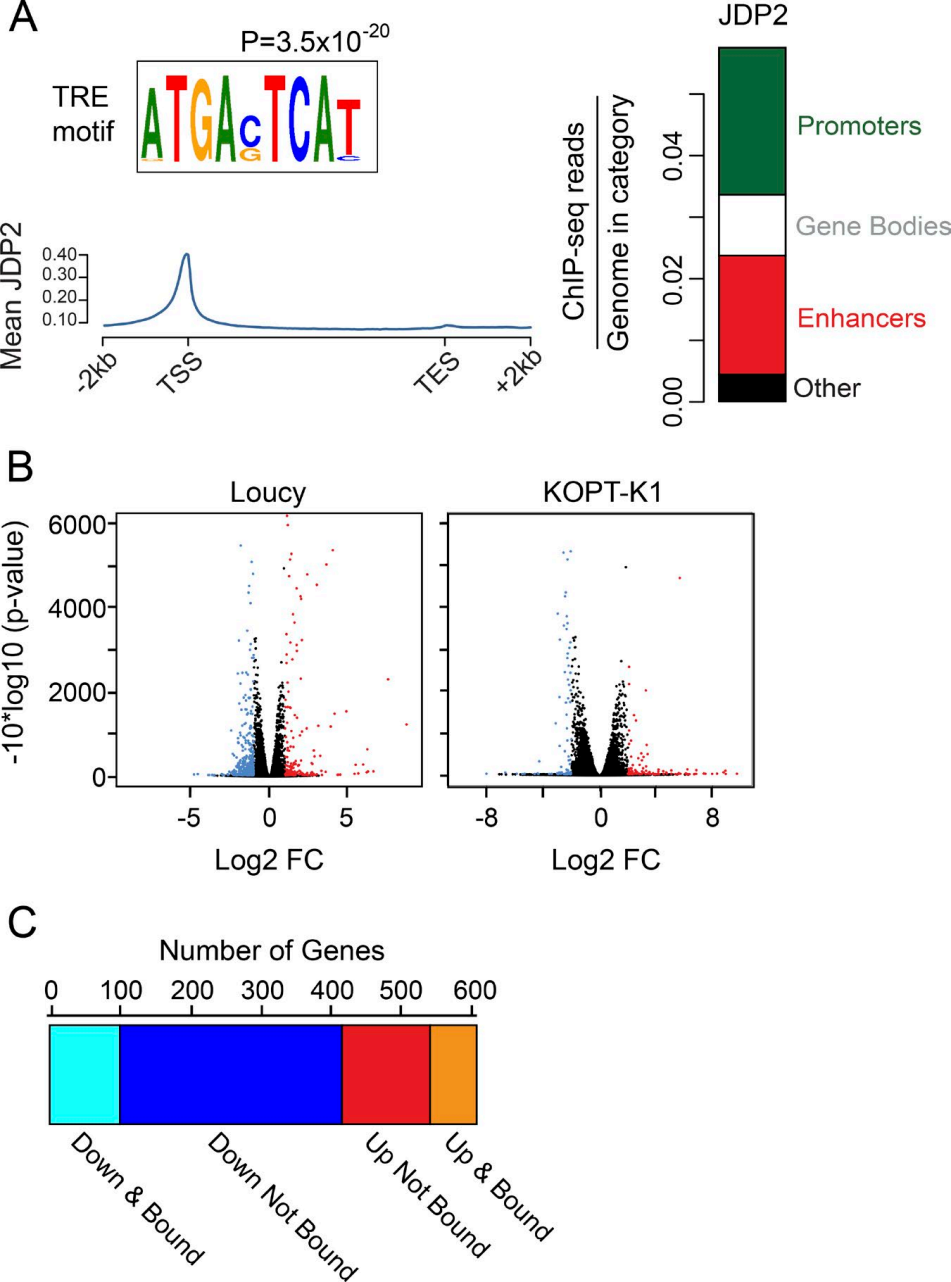
**JDP2 occupies both promoters and enhancers and can both activate and repress target genes. (A)** The preferred binding sequence for JDP2 is the palindromic TRE motif as calculated using Analysis of Motif Enrichment from the MEME suite. The adjusted P value is shown. Mean JDP2 occupancy from the TSS to TES at gene bodies, as analyzed from genome-wide binding by ChIP-seq in Loucy cells. The y axis shows ChIP-seq read concentration normalized to number of base pairs in the given region type. Right: Relative genome-wide occupancy of JDP2 at gene promoters (green), at enhancers (red), and within the gene body (white). **(B)** Volcano plot showing log2 fold change (FC) in gene expression 20 h after JDP2 knockdown in Loucy (left) and KOPT-K1 (right) cells stably expressing a doxycycline-inducible JDP2 shRNA. The y axis shows adjusted P value. Genes represented in blue are significantly down-regulated, those in red are significantly up-regulated, and those in black are not differentially expressed. RNA-seq was performed in biological triplicate. **(C)** Graph illustrating the number of genes in Loucy cells that are differentially expressed (by RNA-seq) after JDP2 knockdown and either bound by JDP2 (by ChIP-seq; direct target genes) or not bound by JDP2 (genes regulated indirectly after JDP2 knockdown).

To gain insight into the transcriptional role of JDP2, we performed chromatin immunoprecipitation (ChIP) with massively parallel sequencing (ChIP-seq) in Loucy cells. Analysis of favored binding motifs identified a significant preference for JDP2 binding at the palindromic TRE sequence ATGA[C/G]TCAT (corrected P value = 3.5 × 10^−20^; Fig. 3 A), a sequence previously demonstrated to bind JDP2 in functional experiments (Weidenfeld-Baranboim et al., 2008; Jolma et al., 2013). Interestingly, there was no enrichment for JDP2 binding to the cAMP response element (CRE) sequence ATGACGTCAT (corrected P value = 1). JDP2 occupied both active promoters and enhancers, as illustrated by its cooccupancy with histone H3 lysine 27 acetylation (H3K27ac; Fig. 3 A). The fact that JDP2 binding correlates with H3K27Ac modification indicates that JDP2 binding is likely affecting the expression levels of expressed genes. We performed RNA-seq shortly after JDP2 knockdown in KOPT-K1 and Loucy cells and, despite modest knockdown, detected a similar number of differentially up-regulated and down-regulated genes after JDP2 depletion (in KOPT-K1, 102 significantly up-regulated and 95 significantly down-regulated; in Loucy, 192 significantly up-regulated and 417 significantly down-regulated; Fig. 3 B). Approximately 25% of differentially expressed genes were also bound by JDP2, suggesting these were direct transcriptional targets of JDP2, and that JDP2 may participate in complexes that function either as a transcriptional activators or transcriptional repressors of its direct target genes (Fig. 3 C).

### JDP2 directly regulates MCL1 expression in T-ALL

**Figure 4.**
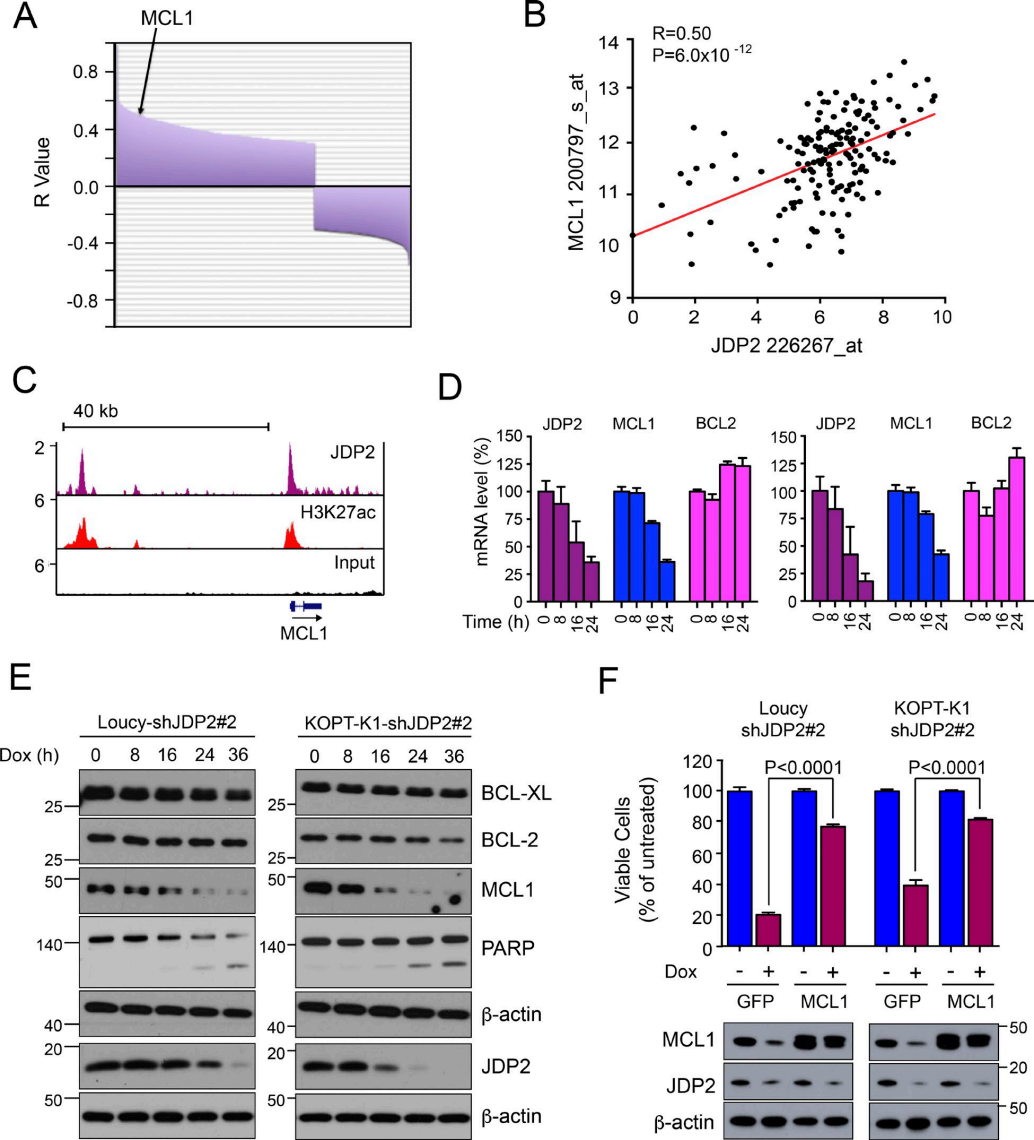
**JDP2 prevents apoptosis by directly regulating *MCL1.* (A)** Waterfall plot showing genes most closely correlated with JDP2, using Pearson correlation analysis of gene expression data from 165 T-ALL patients from the MILE study (Haferlach et al., 2010). The y axis represents the Pearson correlation coefficient (*R* value) positively and negatively associated with JDP2 expression, adjusted for multiple testing using the false discovery rate, and P < 0.001. Genes are distributed across the x-axis, with position of MCL1 highlighted. **(B)** Direct correlation between JDP2 and MCL1, from Fig. 4 A, is shown in detail together with line of best fit. **(C)** ChIP-seq tracks for JDP2 and H3K27ac at the *MCL1* locus in Loucy cells. **(D)** Relative *JDP2*, *MCL1*, and *BCL2* mRNA levels over time after the addition of doxycycline to Loucy cells (left) and KOPT-K1 cells (right) stably expressing doxycycline-inducible *JDP2* shRNA#2. qPCR experiments were performed in triplicate and verified in two independent experiments. **(E)** Western blots showing kinetics of MCL1 depletion and PARP1 cleavage after the addition of doxycycline in Loucy and KOPT-K1 cells expressing a doxycycline-inducible JDP2 shRNA. Representative Western blot from two separate experiments is shown. **(F)** Doxycycline-inducible *JDP2* shRNA expressing Loucy and KOPT-K1 cells were stably transduced to express either GFP (control) or MCL1, and then treated with or without doxycycline, and viable cell number was measured by Cell Titer Glo at 24 h. Bottom: Western blots showing MCL1 and JDP2 expression in the transduced cell lines. P values were calculated using two-tailed Student’s *t* test applied to triplicate experiments performed twice. Data points represent the mean ± standard error of the mean.

We postulated that the dramatic and rapid induction of apoptosis on knockdown of *JDP2* might occur through its ability to directly regulate one or more anti-apoptotic genes. To investigate such an association in primary T-ALL samples, we performed correlation analysis to identify genes whose expression was most closely related to that of *JDP2*, using expression data from 165 T-ALL cases in the MILE study (Table S1; Haferlach et al., 2010). There was a striking positive correlation between *MCL1* mRNA expression and that of *JDP2* (Fig. 4, A and B; Pearson coefficient 0.50, P = 6 × 10^−12^), an association that we were able to validate in three independent T-ALL cohorts (Fig. S4).

To address the possibility that JDP2 directly regulates MCL1, we analyzed our ChIP-seq data and identified JDP2 binding and H3K27ac enrichment at the *MCL1* promoter, as well as at a putative *MCL1* enhancer site 34 kb upstream of the gene (Fig. 4 C) that resides within the same insulated neighborhood as *MCL1* (Hnisz et al., 2016). Consistent with a direct regulatory role, knockdown of *JDP2* in stable cell line clones expressing a doxycycline-inducible *JDP2* shRNA#2 was associated with a marked reduction of *MCL1* mRNA expression (Fig. 4 D), as well as a concomitant reduction in MCL1 protein expression, with simultaneous induction of PARP1 cleavage (Fig. 4 E). Furthermore, when we engineered Loucy and KOPT-K1 cells to stably express *MCL1* such that it was no longer under the transcriptional control of JDP2, the adverse effects on cell viability after JDP2 depletion were significantly, albeit partially, rescued (Fig. 4 F). Together, these data indicate that JDP2 maintains T-ALL cell survival by up-regulating MCL1 through direct transcriptional regulation.

### JDP2 collaborates with c-Myc to initiate T-ALL in the zebrafish

**Figure 5.**
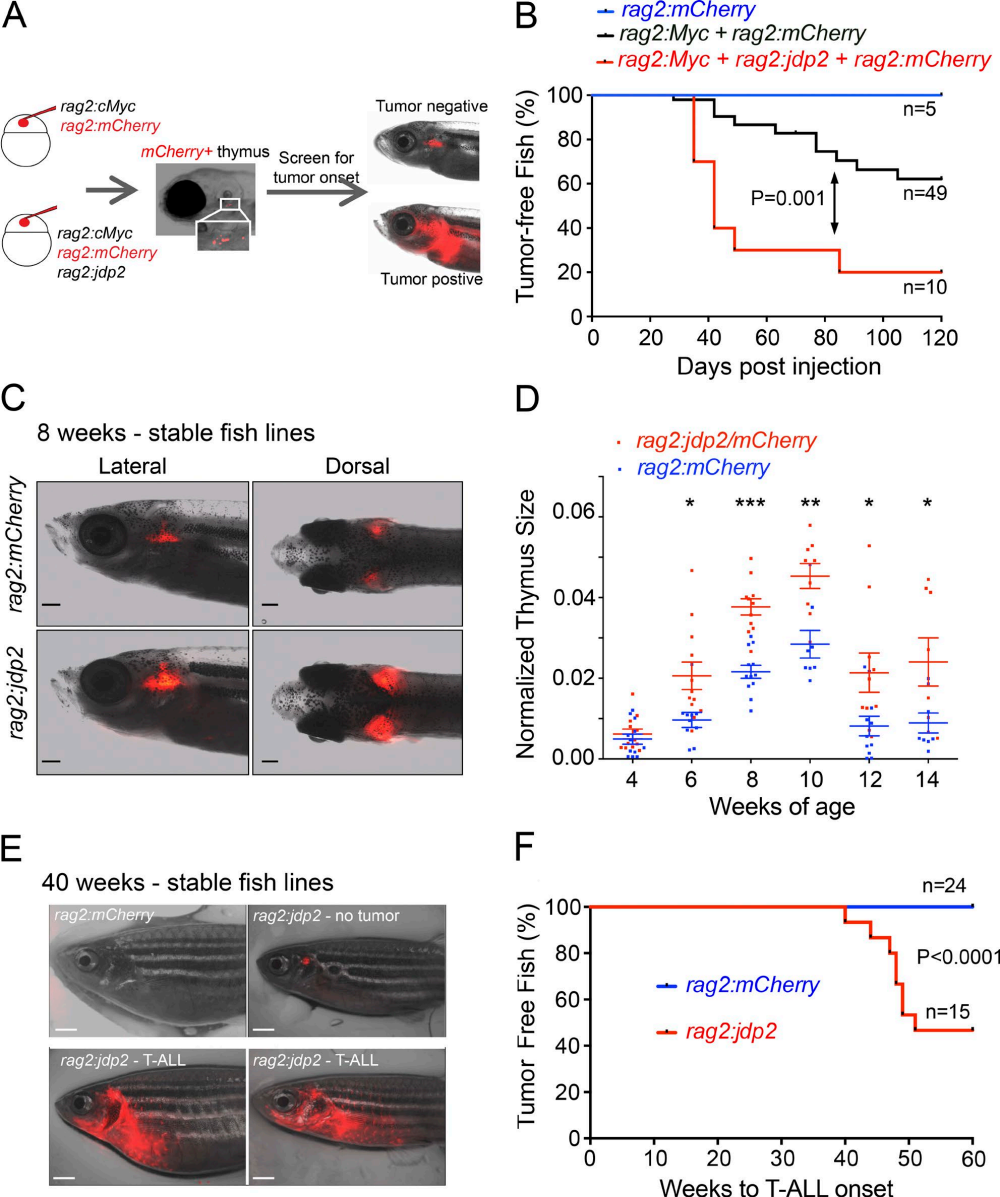
***Jdp2*collaborates with *Myc* and can initiate T-ALL in a zebrafish model. (A)** Schematic showing the coinjection strategy used to identify Myc collaborating genes in first generation mosaic transgenic zebrafish. **(B)** Kaplan–Meier curves showing tumor onset in first-generation mosaic zebrafish coinjected with constructs expressing *rag2:Myc + rag2:jdp2 + rag2:mCherry* (red line), versus *rag2:Myc + rag2:mCherry* versus (black line), versus *rag2:mCherry* alone (blue line). **(C)** Representative fluorescent images of thymuses from 8-wk-old stable transgenic zebrafish expressing *Tg(rag2:mCherry)* and *Tg(rag2:jdp2).* Bars, 1 mm. **(D)** Quantification of thymus size from *Tg(rag2:mCherry)* and *Tg(rag2:jdp2)* assessed by fluorescent microscopy biweekly from 4 to 14 wk of age. To account for intervariation in fish size, thymus size was normalized to head size. * P<0.05; ** P<0.005; *** P<0.0005. Data points represent the mean ± standard error of the mean. **(E)** Representative fluorescent microscopy images from a 40-wk-old *Tg(rag2:mCherry),* and three *Tg(rag2:jdp2)* zebrafish. Bars, 1 mm. **(F)** Kaplan–Meier curves showing tumor onset in stable Mendelian transgenic zebrafish expressing *Tg(rag2:mCherry;* red line) and *Tg(rag2:jdp2;* blue line).

Our data in cell lines convincingly indicate a role for JDP2 in maintaining tumor cell survival, but its ability to initiate T-ALL remained in question. In zebrafish, the murine *c-Myc* oncogene initiates a highly aggressive T-ALL when expressed from the zebrafish *rag2* promoter (Langenau et al., 2003). In this model, transgenes form concatemers before genomic co-integration, enabling mosaic expression of up to three transgenes injected simultaneously into recently fertilized one-cell zebrafish embryos (Langenau et al., 2008). This allows putative oncogenes to be rapidly assessed for their ability to collaborate with c-Myc in transforming early thymocytes. We thus coinjected single-cell embryos with *rag2:mCherry*, *rag2:mCherry/rag2:Myc*, or *rag2:mCherry/rag2:Myc/rag2:jdp2* and monitored the fish by fluorescent microscopy for tumor onset (Fig. 5 A). Coexpression of *jdp2* with murine *c-Myc* led to a marked increase in tumor penetrance, with 80% of fish exhibiting T-ALL at 120 d, compared with 40% when *c-Myc* was expressed alone (P = 0.001; Fig. 5 B). These data are consistent with the high incidence of activating *Jdp2* viral integrations that are selected based on their ability to accelerate the onset of T-ALL in mice with a *c-Myc* transgenic background (Stewart et al., 2007).

### JDP2 can initiate T-ALL in zebrafish lacking ectopic c-Myc expression

Next, we asked whether *jdp2* overexpression was sufficient to initiate tumorigenesis in the zebrafish without the requirement for ectopic coexpression of *c-Myc.* For these experiments, we created a stable founder zebrafish line expressing *rag2:mCherry/rag2:jdp2* (termed here *Tg(rag2:jdp2)*). During normal maturation of the thymus in zebrafish, the size of the organ peaks at 10 wk of age, followed by progressive thymic involution. Similar to other species such as rodents and humans, we noted considerable variations in thymic size between individual zebrafish of the same age (Paton and Goodall, 1904; Nasseri and Eftekhari, 2010). However, in transgenic fish stably expressing *jdp2*, there was a marked increase in thymic size compared with *Tg*(*rag2:mCherry)* fish, which was most apparent at 8–10 wk of age and was associated with a delay in thymic involution when monitored over 14 wk, consistent with hyperplasia (Fig. 5, C and D; and Fig. S5 A). Although thymic fluorescence was undetectable in all *Tg(rag2:mCherry)* fish at 40 wk of age (*n* = 24), it was retained in all *Tg(rag2:jdp2)* fish (*n* = 15; Fig. 5 E). This phenotype is reminiscent of what we had previously observed in transgenic *Tg(rag2:bcl2)* fish, suggesting that jdp2 may play a role in mediating anti-apoptotic signaling (Feng et al., 2010).

**Figure 6.**
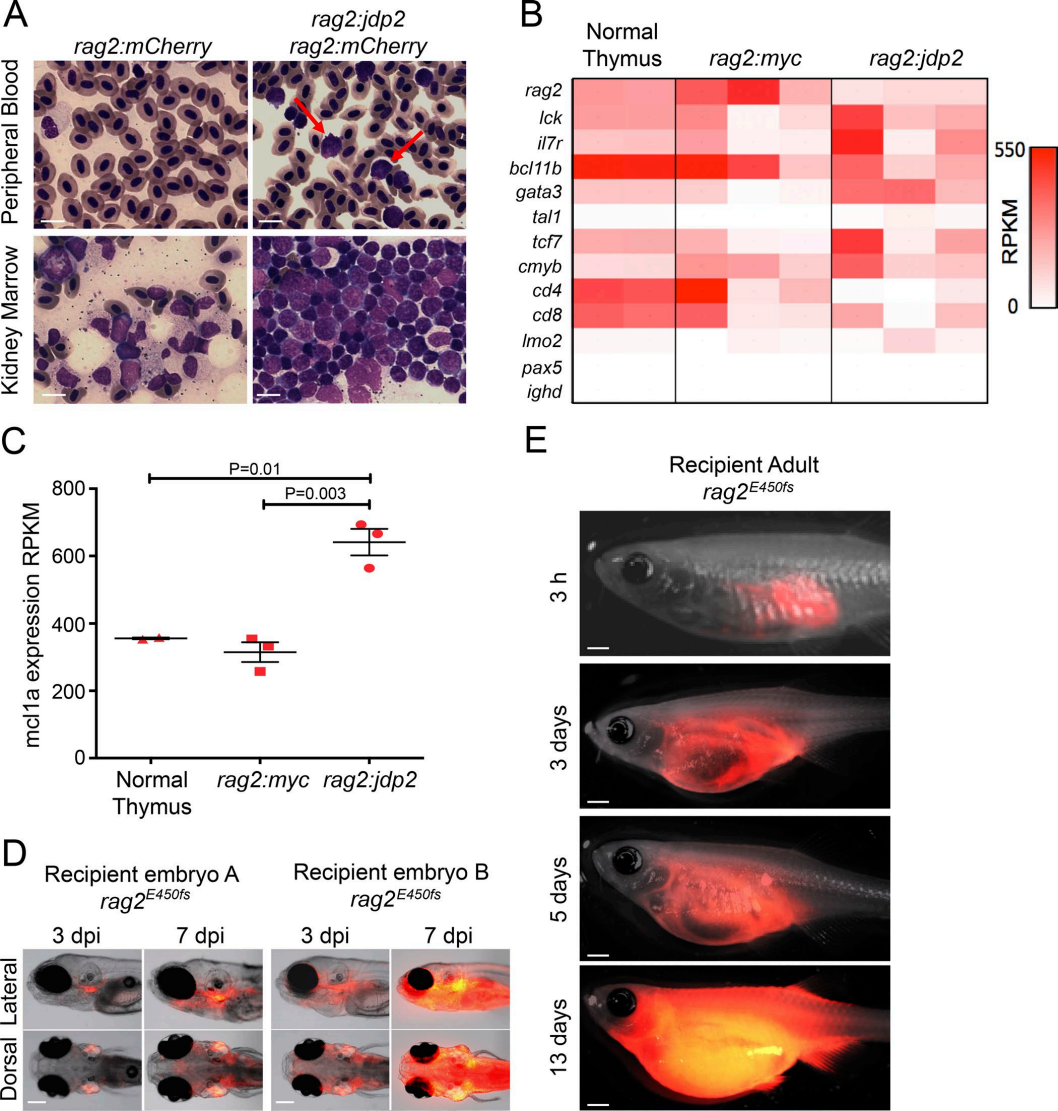
***Jdp2*initiates a transplantable T-ALL when expressed from the *rag2* promoter. (A)** Hematoxylin and eosin staining of peripheral blood and kidney marrow smears from 11-mo-old *Tg(rag2:mCherry)* and *Tg(rag2:jdp2)* fish. Note that red blood cells are nucleated in normal zebrafish. Black arrow identifies a mature myeloid cell, and red arrows, circulating blast cells. Bars, 10 µm. **(B)** Heatmap showing expression of selected genes from FACS-purified cells from normal thymocytes from *Tg(rag2:mCherry)* zebrafish (each column *n* = 10 pooled), and tumors from *Tg(rag2:jdp2)* and *Tg(rag2:Myc)* fish (each column represents an individual fish), as determined by RNA-seq. RPKM, reads per kilobase of exon per million fragments mapped. **(C)** *mcl1a* expression by RNA-seq analysis of FACS-purified fluorescent cells. For the *Tg(rag2:Myc)* and *Tg(rag2:jdp2)* genotypes, each data point represents an individual fish, whereas for normal thymus, each point represents pooled cells harvested from 10 *Tg(rag2:mCherry)* animals. **(D)** Tumor cells were harvested from *Tg(rag2:jdp2)* fish and injected into the circulation of 2-d-old recipient fish homozygous for a hypomorphic *rag2* allele (E450fs). Two representative embryos are shown, and findings were validated in two independent experiments. Bars, 0.1 mm. **(E)** Tumor cells harvested from *Tg(rag2:jdp2)* fish were injected into the peritoneum of adult homozygous rag2^E450fs^ fish. Recipients were monitored by fluorescence imaging. Findings were validated in three independent experiments. Bars, 0.5 mm.

Strikingly, beginning at 40 wk of age, 8 of 15 *Tg(rag2:jdp2)* fish (53%) developed tumors (Fig. 5 F). Leukemic transformation was indicated by infiltration of mCherry-positive cells beyond the thymus, that progressed to involve the kidney marrow and ultimately the entire fish, similar to previous observations in our *rag2:Myc* transgenic fish lines (Langenau et al., 2003). In *Tg(rag2:jdp2)* fish, the kidney marrow was massively infiltrated with blast cells with a monomorphic appearance and high nuclear-to-cytoplasmic ratio, whereas blast cells were readily detectable on peripheral blood smears, indicative of leukemia (Fig. 6 A). Consistent with a T cell phenotype, qPCR from purified tumor cells showed high *cd3* mRNA expression at levels comparable to those in *Tg*(*rag2:Myc)* transgenic tumors and normal thymocytes (Fig. S5 B). Furthermore, the results of RNA-sequencing (RNA-seq) of sorted tumor cells from *Tg(rag2:jdp2)* fish showed expression of *bcl11b*, *gata3*, *lmo2*, *cd8*, *il7r*, and *rag2*, consistent with a diagnosis of T-ALL. Given the ability of JDP2 to regulate *MCL1* expression in human cells, we analyzed *mcl1a* expression in zebrafish tumors. As shown in Fig. 6 C, *mcl1a* expression was approximately twofold higher in *Tg(rag2:jdp2)* compared with *Tg(rag2:Myc)* tumor cells or normal thymocytes (mean expression, 641 vs. 315 and 356 reads per kilobase of exon per million fragments mapped, P = 0.003 and P = 0.01, respectively Fig. 6 C), consistent with our findings of direct regulation of *MCL1* by JDP2 in human cells.

### Tg(rag2:jdp2) tumors are transplantable into secondary recipients

To test whether tumors from *Tg*(*rag2:jdp2)* zebrafish harbor leukemia-initiating cells, we exploited a recently developed immune-compromised zebrafish line with a *rag2^E450fs^* hypomorphic allele, which is permissive of adoptive transfer of allogeneic zebrafish cells (Tang et al., 2014). When tumor cells harvested from *Tg(rag2:jdp2)* fish were injected into the circulation of 2-d-old *rag2^E450fs^ (Casper)* mutant embryos, they rapidly migrated to and engrafted in the thymus of recipient larvae, consistent with their thymic origin (Fig. 6 D). Within 7 d of implantation, tumors had disseminated throughout the larvae, indicating that donor cells had proliferated in the recipient animals, eventually leading to death of the animals (Fig. 6 D). This finding was corroborated by the rapid tumor expansion visible when *Tg(rag2:jdp2)* tumors were injected into the peritoneum of adult *rag2^E450fs^* mutant fish (Fig. 6 E).

### Tg(rag2:jdp2) thymocytes are resistant to glucocorticoids (GCs)

GCs form the backbone of most chemotherapy regimens for adult and pediatric T-ALL, and early steroid response has proven to be an important prognostic marker (Aricò et al., 1995). Furthermore, overexpression of *MCL1* has been identified as a key regulator of steroid resistance in lymphoid malignancy, through its ability to sequester GC-induced BIM (Wei et al., 2006). However, examining GC responses in vitro has been challenging, as unlike the majority of GC-resistant primary T-ALL cases, T-ALL cell lines frequently have mutations or down-regulation of GC receptors (Geley et al., 1996). We previously demonstrated that the zebrafish thymus undergoes rapid apoptosis after the addition of dexamethasone to the fish water (Langenau et al., 2004). Because of the small size of zebrafish larvae and the ability to accurately quantify thymic size through fluorescent microscopy, this transgenic zebrafish line seemed an ideal model with which to test GC resistance in vivo.

**Figure 7.**
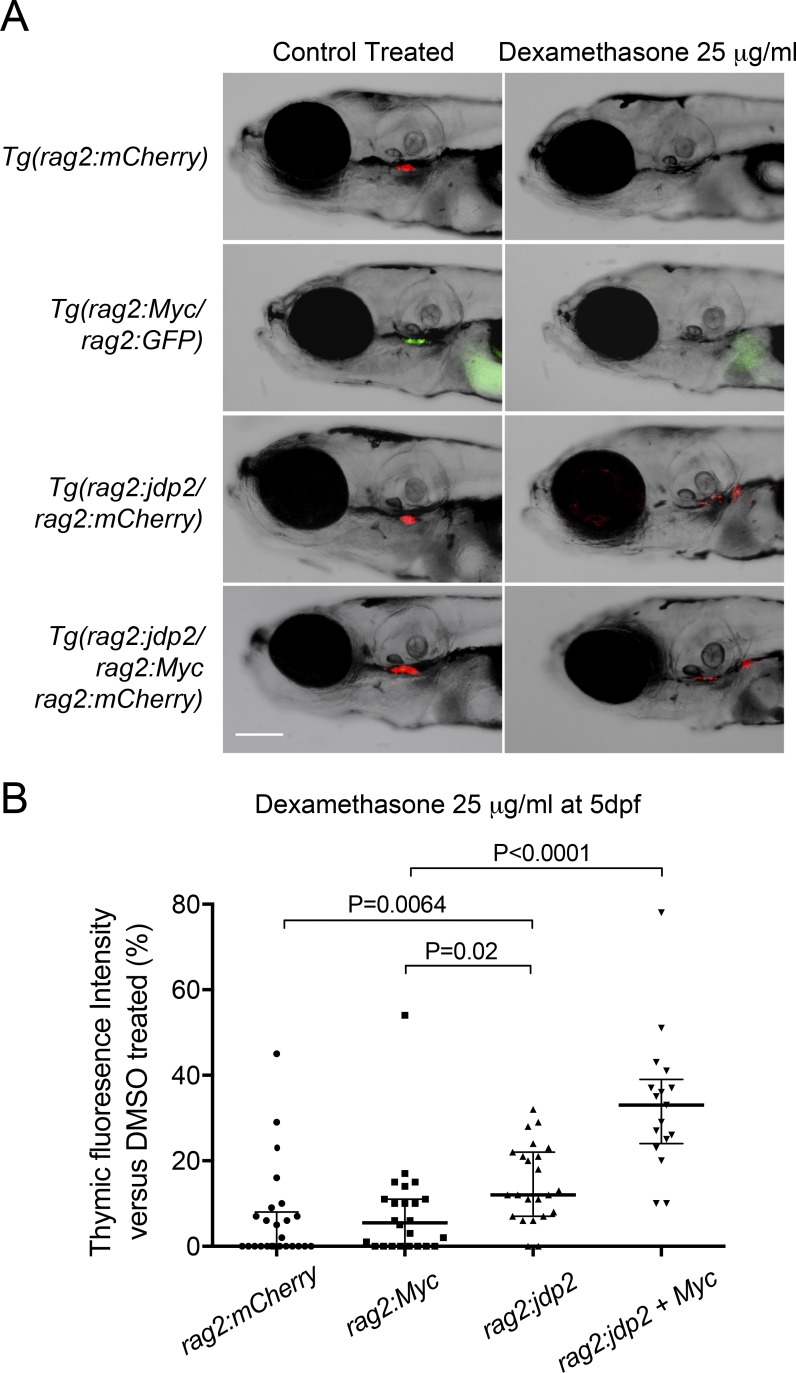
**Thymuses of *Tg(rag2:jdp2)* fish are resistant to dexamethasone. (A)** Representative fluorescent images of thymuses from 8-d-old stable transgenic zebrafish larvae exposed to dexamethasone (25 µg/ml) or DMSO control, from day 5 postfertilization (dpf). Bar, 0.1 mm. **(B)** Quantification of thymic fluorescence in 8-d-old stable transgenic zebrafish larvae exposed to 25 µg/ml dexamethasone or DMSO control from 5 dpf. Values are expressed as a percentage of thymic fluorescence in DMSO control treated zebrafish, and P values were calculated using two-tailed Student’s *t* test. Findings were validated in three independent experiments.

We thus exposed 5-d-old zebrafish to dexamethasone and monitored thymic size. As expected, dexamethasone led to a marked reduction in thymic fluorescence over 3 d of exposure in *Tg(rag2:mCherry)* reporter fish (mean thymic fluorescent intensity 6.6% of vehicle-treated controls) and was also highly effective in *Tg(rag2:Myc)* transgenic fish (mean thymic fluorescent intensity 7.9% of vehicle-treated controls; Fig. 7 A). However, *Tg(rag2:jdp2)* zebrafish had significantly higher amounts of residual thymic tissue after exposure to dexamethasone (mean thymic fluorescent intensity 14.9% of vehicle-treated controls, P = 0.007 and P = 0.02 vs. *rag2:mCherry* and *rag2:Myc*, respectively; Fig. 7, A and B). Fish transgenic for both *Tg(rag2:Myc)* and *Tg(rag2:jdp2)* exhibited the most striking GC resistance (mean thymic fluorescent intensity 33.0% of vehicle-treated control fish; P < 0.0001 compared with the response of *rag2:Myc*). These findings are consistent with our observation that JDP2 directly regulates *MCL1*, which is a known mediator of GC resistance (Wei et al., 2006), thus implicating JDP2-up-regulated MCL1 expression as at least one of the causes of the inferior outcome identified in patients with JDP2 overexpression.

## Discussion

Insertional mutagenesis screens have been highly fruitful in identifying novel oncogenes that subsequently prove to be directly relevant to human disease. For instance, *EVI1*, *HOXA*, and *MEIS1* were first identified by their overexpression resulting from adjacent recurrent integration sites in murine insertional mutagenesis models of AML, before appreciation of their prominent roles in the human disease (Morishita et al., 1988; Nakamura et al., 1996). Similarly, *MYC* and *NOTCH-1*, the most commonly dysregulated oncogenes in human T-ALL, are both targets of retroviral activation in murine insertional models of this disease (Girard et al., 1996; Hoemann et al., 2000; Weng et al., 2004). Interpretation of these assays assumes that integration of viral sequences throughout the genome is random, and that the earliest leukemias arise from cells where the viral LTR has resulted in dysregulation of a nearby tumor suppressor or oncogene. A pertinent example of this phenomenon in humans can be found in the development of T-ALL in severe combined immunodeficiency patients who received retrovirally transduced, gene-corrected autologous hematopoietic stem cells, where the viral LTR integrated into the vicinity of the *LMO2* gene (Hacein-Bey-Abina et al., 2003, 2008; Howe et al., 2008).

Our data strongly support both viral and transposase-based insertional mutagenesis models that implicate JDP2 as a T-ALL oncogene. One proposed mechanism by which the JDP2 protein exerts its oncogenicity in this setting is through suppression of *TP53*, given that T-ALLs arising on a *TP53* heterozygous background have a particularly high frequency of insertions at the *Jdp2* promoter (van der Weyden et al., 2013). Although this mechanism is intriguing, it is unlikely to be responsible for the ability of *jdp2* to transform thymocytes in our zebrafish model, where loss of *tp53* neither induces T-ALL nor collaborates with Myc in tumorigenesis (Gutierrez et al., 2014). *jdp2* thus represents one of only a few select oncogenes (including *Myc*, *Notch*, and *Myr-AKT*) capable of initiating T-ALL in the zebrafish; the long disease latency and incomplete penetrance in our model suggests that as-yet undiscovered secondary mutations are likely to be involved in transformation (Langenau et al., 2003; Gutierrez et al., 2011; Blackburn et al., 2012, 2014).

Although the majority of studies support a repressive role for JDP2 through its ability to recruit histone deacetylases and its interaction with histones and the PRC2 complex, our ChIP-seq data suggest it can also have a role as a transcription activator in T-ALL cells. Unfortunately, we were unsuccessful in studying histone modifications after JDP2 knockdown, presumably because of nuclear fragmentation of cells undergoing apoptosis. Interestingly, a previous study has shown that JDP2 can strongly enhance transcription from promoters containing TREs, but not from those containing CREs, consistent with the preferential binding of JDP2 to TREs at active genes identified in our ChIP-seq experiments (Weidenfeld-Baranboim et al., 2008). One other factor that could potentially impact the transcriptional activity of JDP2 is that approximately half of ETP-ALL cases have inactivating mutations of members of the PRC2 complex (Zhang et al., 2012). Loucy cells, for instance, lack detectable *EZH2* mRNA expression, but whether this influences the role of JDP2 as a repressor or activator will require further study.

The most striking phenotype in vitro was a dependency of human T-ALL–derived cell lines on JDP2 for their survival. Mechanistically, JDP2 regulates prosurvival signaling through direct transcriptional regulation of the anti-apoptotic gene *MCL1*; indeed, programmed expression of *MCL1* was sufficient to rescue the apoptotic effects of JDP2 depletion. We and others have recently shown that ETP-ALL cells depend on BCL2 for their survival (Anderson et al., 2014; Chonghaile et al., 2014; Peirs et al., 2014). Given that BCL2 and MCL1 are able to bind and sequester the same BH3-only proteins, BIM and BID, our current data suggest that Loucy cells are “primed” for apoptotic death through this pathway, such that inhibition of either BCL2 or MCL1 is sufficient to trigger apoptosis. In support of this concept, previous studies have shown that MCL1 depletion alone dramatically impairs the viability of Loucy cells (Ariës et al., 2013).

Many ALL cell lines show down-regulation or mutations of the GC receptor, features that are only rarely found in primary T-ALL samples, limiting what can be learned from studying GC sensitivity of T-ALL cell lines in vitro (Geley et al., 1996). This was one of the reasons we chose to study GC responses in our zebrafish model, where the ease in imaging the fluorescently labeled thymus in zebrafish embryos, and its exquisite sensitivity to dexamethasone, makes it an attractive model for studying GC responses in vivo. We show that the premalignant thymus in *Tg(rag2:jdp2)* transgenic zebrafish is resistant to GC treatment. Similar to our findings in T-ALL cell lines and patients, zebrafish *mcl1* expression levels in thymocytes from *Tg(rag2:jdp2)* were approximately twice those measured in wild-type or *Tg(rag2:Myc)* fish. In light of *MCL1* as an important mediator of GC resistance in lymphoid cells (Wei et al., 2006), these observations provide a potential mechanistic link between *JDP2* overexpression, GC resistance, and inferior survival in patients with T-ALL. We have shown that JDP2 is preferentially up-regulated in ETP-ALL (Fig. 1, C and D), and support for increased GC resistance in human ETP-ALL is provided by recent studies in human T-ALL patient-derived tumor graft models, in which Delgado-Martin et al. (2017) demonstrated that cells from ETP T-ALL models are intrinsically more resistant to GCs than are cells from non-ETP T-ALLs. Given that bZIP proteins such as JDP2 are extremely challenging to target directly, our data suggest that inhibiting its downstream effector MCL1 would be a rationale alternative approach in patients with *JDP2* overexpression, once specific and potent MCL1 inhibitors become available for clinical use.

## Materials and methods

### Cell lines

The identity of T-ALL cell lines used in this study was verified by short tandem repeat analysis using the PowerPlex 1.2 system (Promega) in January 2013. T-ALL cells were maintained in RPMI-1640 supplemented with 10% FBS, l-glutamine, and penicillin/streptomycin. HEK-293T cells were recently obtained from American Type Culture Collection and maintained in DMEM supplemented with 10% FBS, l-glutamine, and penicillin/streptomycin.

### shRNA knockdown experiments in T-ALL cell lines

pLKO.1-puromycin lentiviral vectors expressing shRNAs targeting JDP2 or control (luciferase and GFP) were obtained from the RNAi Consortium of the Broad Institute. JDP2 shRNA#1 equates to clone TRCN0000019001 (targeting 5′-ACTCATGAACGCAGAGCTGAA-3′), and JDP2 shRNA#2 equates to clone TRCN0000019002 (targeting 5′-CGAGTCAGAAGGCAACCCACT-3′). Production of lentivirus and viral transductions was performed as previously described (Sanda et al., 2012). To create doxycycline-inducible JDP2 knockdown KOPT-K1 and Loucy cells, JDP2shRNA#2 oligonucleotides were annealed and cloned into the AgeI/EcoRI sites of pLKO1-Tet-on (upper, 5′-CCGGCGAGTCAGAAGGCAACCCACTCTCGAGAGTGGGTTGCCTTCTGACTCGTTTTT-3′; lower, 5′-AATTAAAAACGAGTCAGAAGGCAACCCACTCTCGAGAGTGGGTTGCCTTCTGACTCG-3′). KOPT-K1 and Loucy cells were transduced with lentiviral supernatants, selected by puromycin, and single-cell cloned by limiting dilution. Single-cell clones were expanded and assessed for shRNA inducibility by qPCR and Western blotting for JDP2, 24 h after the addition of doxycycline. To generate a retrovirus vector encoding a wobble mutant JDP2 (JDP2w2, where JDP2w2 refers to JDP2 cDNA containing silent/wobble mutations (underlined) in the target site of shRNA#2, 5′-CGAATCGGAGGGTAATCCACT-3′), human JDP2 cDNA was amplified by PCR using forward primer, 5′-TTGGGATCCGCCACCATGATGCCTGGGCAGATCCC-3′, and reverse primer, 5′-TATAGTCGACTCACTTCTTCTCGAGCTGCTCGAGCAGTGGATTACCCTCCGATTCGGGGGT-3′. The PCR products were cut by BamHI and SalI and cloned into MSCV-IRES-GFP vector between BamHI and XhoI sites. For rescue experiments, KOPT-K1 cells were stably transduced with MSCV-IRES-GFP or MSCV-JDP2w2-IRES-GFP, sorted for GFP expression by FACS, and subjected to knockdown experiments with pLKO1-JDP2sh#2-puro vector. Cell growth assays were performed in 96-well plate format, and cell number was assessed at 72 h by Cell Titer Glo (Promega). The retroviral vector encoding MCL1 was generated as previously described (Akahane et al., 2016).

### Apoptosis assays

TUNEL assay and propidium iodide (PI) staining were performed using the ApopTag Fluorescein Direct In Situ Apoptosis Detection Kit (EMD Millipore) according to the manufacturer’s recommendation. Briefly, 2 × 10^6^ cells of each treated sample were fixed with 1% paraformaldehyde in PBS for 15 min on ice, washed in PBS, and incubated with 70% ethanol at −20°C overnight. The cells were then washed and incubated with DNA labeling solution containing TdT and BrdU triphosphates for 4 h at 37°C. The cells were washed and incubated in the staining buffer containing an Alexa Fluor 488 dye–labeled anti-BrdU antibody for 30 min at room temperature, and a mixture of PI/RNase was added. After 30-min incubation at room temperature, TUNEL positivity and cell cycle distribution were analyzed by FACSCalibur (BD Biosciences). Annexin V and PI double staining was also used for detecting apoptosis. 2 × 10^5^ cells of each treated sample were washed with PBS, incubated in staining buffer containing FITC-conjugated anti–annexin V antibody and PI (MBL International), and then analyzed by FACSCalibur.

### T-ALL patient samples

Gene expression data were analyzed from published Affymetrix U133 Plus 2.0 arrays from pediatric patients treated on the COG P9404 trial and from HumanHT-12 v4 Expression BeadChip (Illumina) from 53 adult T-ALL patients treated on the E2993 ECOG trial (Gutierrez et al., 2010; Van Vlierberghe et al., 2013). qPCR for *JDP2* was performed from 34 diagnostic adult T-ALL cases from the UKALL14 trial. Informed consent was obtained from all patients according to the Declaration of Helsinki, and the trial was approved by the West London Research Ethics Council (09/H0711/90).

### Normal thymic subsets

Thymuses were obtained as surgical tissue discards from children aged 7 wk to 3 yr undergoing cardiac surgery at the Erasmus MC Rotterdam, with informed consent from the parents. The children did not have immunological abnormalities. Thymocytes were isolated by cutting the thymic lobes into small pieces and squeezing them through a metal mesh, then stored in liquid nitrogen. Thymocytes from five donors were used to reduce intersample variation. After thawing, pooling, and Ficoll density separation, thymocytes were labeled with fluorochrome-conjugated monoclonal antibodies. For the isolation of CD34^+^CD1a^−^ and CD34^+^CD1a^+^ thymocytes, CD34^+^ progenitor cells were prepurified by AUTOMACS (Miltenyi Biotec) using the CD34 microbeads kit UltraPure (Miltenyi Biotec) before FACS sorting. The following monoclonal human antibodies were used: CD1a-PE (HI149), CD3-APC (SK7), CD4-Pacific blue (RPA-T4), CD8-FITC (SK1), CD13-PE (L138), CD16-PE (Leu11c), CD19-PE (4G7), CD33-PE (P67.6), CD34-APC (581), CD34-PE (8G12), and CD56-PE (MY31; all BD Bioscience). Cell sorting was performed on a FACSAria II cell sorter (BD Biosciences). All populations were directly sorted as indicated by their marker expression (Dik et al., 2005).

### RNA extraction and cDNA and expression analysis

mRNA was extracted using the RNeasy Mini Kit (Qiagen). Purified RNA was reverse-transcribed with the Superscript III RT kit (Invitrogen). qPCR was performed with the Applied Biosystems ViiA 7 using gene-specific primers and SYBR Green PCR Master Mix (Roche). Primer sequences for qPCR were as follows: human JDP2-F 5′-ACGGAGTTTCTGCAGCGG-3′; human JDP2-R 5′-CAGCATCAGGATGAGCTGC-3′; human β-actin-F 5′-AGAGCTACGAGCTGCCTGAC-3′; human β-actin-R AGCACTGTGTTGGCGTACAG-3′; human GAPDH 5′-TGCACCACCAACTGCTTAGC-3′; human GAPDH 5′-GGCATGGACTGTGGTCATGAG-3′; human MCL1-F 5′-GGACAAAACGGGACTGGCTA-3′; and human MCL1-R 5′-TGCCAAACCAGCTCCTACTC-3′.

### Western blots and antibodies

Whole cell lysates were prepared in RIPA buffer. For JDP2 Western blots, nuclear fraction protein was prepared using a Nuclear Extract Kit (Active Motif). Protein concentration was quantified with a Pierce BCA Protein Assay Kit (Thermo Fisher Scientific). Equivalent amounts of protein were diluted in the Laemmli sample buffer (Bio-Rad Laboratories) and separated by SDS-PAGE. Proteins were transferred to PVDF membranes (Millipore) and subjected to immune blot analysis with specific antibodies for JDP2 (ab40916; Abcam), BCL2 (4223S; Cell Signaling Technology), BCL-XL (2764; Cell Signaling Technology), MCL1 (5453; Cell Signaling Technology), PARP1 (9542P; Cell Signaling Technology), cleaved caspase 3 (9664S; Cell Signaling Technology), and β-actin (4967S; Cell Signaling Technology). All primary antibodies were diluted at 1:2,000 in 5% milk in PBST (0.5% Tween-20 in PBS), except for β-actin, which was diluted to 1:5,000 in 5% milk in PBST.

### ChIP-seq experiments

ChIP coupled with massively parallel DNA sequencing (ChIP-seq) was performed as previously described (Lee et al., 2006; Marson et al., 2008; Mansour et al., 2014). Antibodies used for ChIP were anti-JDP2 (sc-23456X; Santa Cruz Biotechnology) and anti-H3K27ac (ab4729; Abcam). For each ChIP, 10 µg antibody was added to 3 ml of sonicated nuclear extract. Illumina sequencing and library construction methods were previously described (Marson et al., 2008). Reads were aligned to build hg19 of the human genome using Bowtie 1.0.1 with parameters –best –k 2 –m 2–sam –l 40 (Langmead et al., 2009). For visualization in the UCSC genome browser (Kent et al., 2002), WIG files were created from aligned ChIP-Seq read positions using MACS with parameters –w –S –space = 50 –nomodel –shiftsize = 200 to artificially extend reads to be 200 bp and calculate their density in 50-bp bins (Zhang et al., 2008). Read counts in 50-bp bins were then normalized to the millions of mapped reads, giving reads per million values.

### ChIP-seq processing

Regions enriched in ChIP-seq signal, termed islands, were identified using SICER with corresponding control and parameters –t 1 (reads with unique positions) –w 200 (window size 200 bp) –g 200 (gap size 200 bp) –i 200 (read shift 200 bp) –t 0.74 (mappable genome) –p 1e-3 (island significance threshold; Zang et al., 2009). We used SICER over other peak callers to capture the observed range in type/breadth of islands.

### Read distribution in regions

To determine the preference of JDP2 to bind different types of regions, we counted the reads from JDP2 ChIP-Seq in promoters (TSS ± 1,000 bp), gene bodies (TSS + 1,000 to transcription end site [TES]), and enhancers (H3K27ac peaks). Only genes greater than 1,000 bp were considered. Reads were first counted in promoters; remaining reads were counted in bodies; remaining reads were counted in enhancers. Counts were divided by the total number of bases in these region types and displayed as percentage stacked bar plots. The mean gene-centric profile of JDP2 was determined using bamToGFF (https://github.com/BradnerLab/pipeline) on three regions. The upstream 2,000 bp was divided into 50 equally sized bins; the region TSS to TES was divided into 150 equally sized bins; the downstream 2,000 bp was divided into 50 equally sized bins. Each BamToGFF produced matrices of RPM-normalized ChIP-Seq read counts in these bins and used parameters –e 200 –f 0 –r. The mean value in each bin was plotted using Matplot. ChIP-Seq signal around individual genomic landmarks (promoters, JDP2 islands, and enhancers) was calculated using BamToGFF and is displayed in Fig. 3 A. Promoters (TSS ± 5,000 bp), JDP2 islands (10,000 bp centered on the middle of JDP2 islands from SICER), and enhancers (10,000 bp centered on the middle of H3K27ac islands) were partitioned into 50, 50, and 150 bins, respectively. Heatmaps represent the RPM-normalized read counts in these bins in each of 37,973 promoters of RefSeq genes or 27,643 H3K27ac islands that do not contact a promoter or 12,649 JDP2 islands. Each region is a row, and rows are ordered by the sum of JDP2 signal in that row.

### Motif analyses

To calculate the enrichment of JDP2 DNA-binding motifs in JDP2 islands, we used AME from the MEME suite (McLeay and Bailey, 2010). Position weight matrices were used to scan the genome sequence in the JDP2 islands with parameters –method fisher –scoring totalhits to calculate the count enrichment in these motifs (Jolma et al., 2013). The corrected P value is reported.

### Zebrafish models

Zebrafish maintenance and all animal experimental procedures were approved by the Dana-Farber Cancer Institute Institutional Animal Care and Use Committee–approved protocol #02-107 and Massachusetts General Hospital Subcommittee on Research Animal Care, under protocol #2011N000127.

### Mosaic coinjection experiments

DNA constructs used to generate mosaic transgenic zebrafish included *rag2:mCherry* and *rag2:mMyc* (encoding murine Myc; Langenau et al., 2003; Smith et al., 2010). Zebrafish *jdp2b* (corresponding to ENSDARG00000020133; zgc:92851) was amplified from zebrafish cDNA (primer forward: 5′-CACCATGATGCCTGGTCAAATCCCTGATCC-3′; primer reverse: 5′-TCAGTCTTCGCGGGGCTCCAGC-3′), subcloned into pENTR gateway system (Life Technologies) and transferred into the *rag2* promoter destination vector using LR clonase II (Life Technologies). Plasmids were linearized with NotI or XhoI and purified. Mosaic transgenic animals were generated as previously described (Langenau et al., 2008). 40 ng/µl *rag2:mCherry* was mixed with 40 ng/µl *rag2:Myc*, and 40 ng/µl *rag2:jdp2* was microinjected into one-cell-stage Tu/AB embryos. Mosaic transgenic animals were monitored for T-ALL onset at day 21 and every 7 d thereafter by fluorescent microscopy after tricaine anesthesia.

### Generation of stable *Tg(rag2:jdp2/rag2:mCherry)* zebrafish line

To create a stable *Tg(rag2:jdp2/rag2:mCherry)* zebrafish line, *jdp2b* (corresponding to zebrafish ENSDARG00000020133; zgc:92851) was amplified from zebrafish cDNA (primer forward: 5′-TATAGGATCCGCCCAAATCCCTGATC-3′; primer reverse 5′-TATAATCGATTCAGCGGGGCTCCAGC-3′), digested by BAMHI and ClaI, and cloned into the BAMHI/ClaI sites downstream of the zebrafish *rag2* promoter in a modified pBluescript vector containing flanked recognition sequences for I-SceI meganuclease (Gutierrez et al., 2011). *rag2:mCherry* and *rag2:jdp2* vectors were digested by I-SceI and coinjected together into one-cell-stage AB embryos using a previously described I-SceI coinjection strategy (Grabher and Wittbrodt, 2008). *jdp2b* expression was quantified by isolating mCherry fluorescent cells by FACS, extracting RNA, and performing qPCR with the following primers: zf-jdp2b-F 5′-TTGCAGCTGCTCGCTGTC-3′; zf-jdp2b-R 5′-GCTCCGACTTCAGCTCCTC-3′; zf-β-actin-F 5′-TACAATGAGCTCCGTGTTGC-3′; and zf-β-actin-R 5′-ACATACATGGCAGGGGTGTT-3′). *Tg(rag2:mCherry)* and *Tg(rag2:jdp2/rag2:mCherry)* zebrafish were screened for tumors by fluorescence microscopy every 2 wk starting from the age of 10 wk. Fluorescence microscopy was performed with a Leica M420 microscope equipped with an X-Cite 120 Fluorescence Illumination System (EXFO). Kidney and peripheral blood smears, as well as MGG staining, were performed as previously described (Gjini et al., 2015).

### Imaging and quantification

For in vivo steroid sensitivity testing, 5-dpf larvae were sorted for thymic fluorescence, treated with dexamethasone (25 µg/ml, 0.25% ethanol) or ethanol vehicle alone (0.25%) in standard egg water, and imaged by fluorescent and light microscopy. For bright-field DIC images, a Zeiss Axio Imager.Z1 compound microscope equipped with an AxioCam HRc was used. For live imaging, zebrafish and larvae were anaesthetized using 0.016% tricaine (Sigma) and mounted in 4% methylcellulose (Sigma). A Nikon SMZ1500 microscope equipped with a Nikon digital sight DS-U1 camera was used for capturing both bright-field and fluorescent images from live zebrafish and larvae. For thymic fluorescence quantification, all animals in the same experiments were imaged at the same condition, and the acquired fluorescent images were quantified in ImageJ (NIH) by measuring the fluorescent-covered areas. To account for variation in fish size, the thymic fluorescent area was normalized against the bright-field area of the fish head. Overlays were created using ImageJ and Adobe Photoshop 7.0.1.

### RNA-seq data processing

The RNA-seq data were processed using Tophat and Cufflinks package according the RNA-seq experiments protocol (Trapnell et al., 2012). First, RNA-seq raw reads were filtered using Fastx-toolkit (FASTX-Toolkit, 2018), the reads with *n* > 5% or with low-quality bases (Q <20) >20% were removed, and the remaining low-quality bases (Q <20) were trimmed. The clean reads were aligned using Tophat2 (Kim et al., 2013), zebrafish genome release version Zv9, and the Ensembl annotated version 73 was used as the transcript model reference for the alignment as well as the isoform quantifications. After alignment, the gene and isoform expression levels were produced using Cufflinks in fragments per kilobase of transcript per million fragments mapped. RNA-seq in Loucy and KOPTK1 cells were aligned to the hg19 version of the human reference genome using tophat with parameters –library-type fr-firststrand –no-novel-juncs and -G set to human RefSeq genes downloaded on July 5, 2017. Per-RefSeq-gene expression was calculated using htseq-count with parameters -i gene_id –stranded=reverse -f bam -m intersection-strict (Anders et al., 2015). Differential expression was determined using DESeq2 (Love et al., 2014). Genes were considered significantly differentially expressed if they had a DESeq2-normalized log2 fold-change greater or less than 1 (2 linear fold) and a DESeq2 adjusted P value < 0.05.

### Datasets, accession numbers, and data analyses

The following GEO gene expression datasets were analyzed: GSE14618 (COG P9404 trial), GSE26713 (COALL/DCOG trials), GSE42328 (ECOG2993), and GSE13159 (MILE study; Gutierrez et al., 2010; Haferlach et al., 2010; Homminga et al., 2011; Van Vlierberghe et al., 2013). Loucy input control and H3K27ac ChIP-seq are available under GSM2311760 and GSM2037788, respectively. JDP2 ChIP-seq and RNA-seq datasets are available under GSE115465 and GSE115464, respectively. 

### Statistical analysis

Differences in overall survival for patients on the COG P9404 trial, and differences in time to tumor formation in zebrafish, were assessed by the log-rank test, and time-to-event distributions were estimated and plotted via the Kaplan–Meier method in Prism. For survival data, patients were determined as JDP2 positive if their expression value was in the top quartile. Our use of the top quartile to define T-ALLs with aberrant *JDP2* expression levels is consistent with data from qPCR showing that the top quartile of patients overexpress *JDP2* compared with the ISP thymic subset (Fig. 1 B), which expresses the highest *JDP2* levels among all normal thymic subsets. Differences in *JDP2* mRNA expression between ETP versus non-ETP subgroups were calculated using the Student’s *t* test. Correlation analysis was performed using the R2 database (R2: Genomics Analysis and Visualization Platform; http://r2.amc.nl) from 165 T-ALL patients recruited onto the MILE study (Haferlach et al., 2010), using JDP2 Affymetrix probe 226267_at and a P value cutoff of <0.001 adjusted for multiple testing using the false discovery rate. Waterfall plots were generated in Excel with *R* value cutoffs of ±0.3.

### Online supplemental material

Fig. S1 shows cell growth kinetics after lentivirus-mediated shRNA depletion of JDP2 with two independent shRNAs in Loucy cells and KOPT-K1 T-ALL cells. Fig. S2 shows specificity of selected JDP2 shRNAs through lack of toxicity across a panel of cell lines, lack of effect in non-JDP2-expressing DND-41 cells, and rescue of cell growth with JDP2 reexpression. Fig. S3 shows that JDP2 depletion initiates apoptosis as determined by flow cytometry for annexin V and Western blotting for PARP1 and caspase-3 cleavage. Fig. S4 shows positive correlation of *JDP2* and *MCL1* expression in different T-ALL patient cohorts. Fig. S5 shows delayed involution of thymus in sections from 23-wk-old Tg(rag2:jdp2) zebrafish, as well as expression of CD3 by qPCR. Table S1 shows correlation analysis of genes most negatively and positively associated with *JDP2* expression in T-ALL patients from the MILE study.
